# Recent Southern Hemisphere Lamprimine Stag Beetle in Cretaceous Burmese Amber and Its Biogeographic Implications (Coleoptera: Lucanidae) †

**DOI:** 10.3390/insects15090658

**Published:** 2024-08-30

**Authors:** Yali Yu, Zhenhua Liu, Zhiqiang Li, Chenyang Cai

**Affiliations:** 1 Guangdong Key Laboratory of Animal Conservation and Resource Utilization, Guangdong Public Laboratory of Wild Animal Conservation and Utilization, Institute of Zoology, Guangdong Academy of Sciences, Guangzhou 510260, China; yuyl@giz.gd.cn (Y.Y.); liuzhh_beetle@giz.gd.cn (Z.L.); 2 State Key Laboratory of Palaeobiology and Stratigraphy, Nanjing Institute of Geology and Palaeontology, Chinese Academy of Sciences, Nanjing 210008, China

**Keywords:** Lucanidae, Lampriminae, Burmese amber, biogeographic implications, evolution

## Abstract

**Simple Summary:**

Lucanidae (stag beetles) is a small family and one of the most ancient groups within the Scarabaeoidea superfamily. Most adult lucanids exhibit clear sexual dimorphism, with males often having impressive mandibles that are used in fierce competition for mates. Fossils of Lucanidae from the Mesozoic era are rare. In this study, we describe a new lucanid fossil, *Prostreptocerus burmiticus* Yu & Cai gen. et sp. nov., found in mid-Cretaceous Burmese (Kachin) amber, which shares similar features with the modern Lampriminae. This discovery marks the first fossil record of Lampriminae in mid-Cretaceous amber from northern Myanmar and is the oldest reliable record of the subfamily. The well-developed mandibles and curved claws of *Prostreptocerus* Yu & Cai provide additional evidence for sexual dimorphism and combat behavior in Mesozoic lucanids. This new taxon also enhances our understanding of early biodiversity and the biogeographic implications of stag beetles.

**Abstract:**

A new stag beetle fossil, *Prostreptocerus burmiticus* Yu & Cai gen. et sp. nov., is described based on a single male specimen. This is the first representative of the subfamily Lampriminae (Coleoptera: Scarabaeoidea: Lucanidae) from mid-Cretaceous Burmese amber. The new species is distinctive among Lucanidae due to its well-developed, right-angled mandible, frons featuring a pair of large protuberances, a coarse and sparsely punctate elytral disc, and large tubercles on the humeri. *Prostreptocerus* Yu & Cai is placed within Lampriminae based on several key characteristics. Morphologically, it is most similar to the extant *Streptocerus* Fairmaire, 1850. The current distribution of *Streptocerus* and Lampriminae is primarily restricted to the Southern Hemisphere, suggesting that this lineage is ancient and existed on Gondwanaland, which has significant geographical implications. This discovery extends the fossil record of Lampriminae and provides additional evidence for the existence of sexual dimorphism and potential combat behavior in Mesozoic lucanids. Additionally, *Electraesalopsis* Bai, Zhang & Qiu, 2017, previously placed as Lucanidae *incertae sedis*, shares many characteristics with *Prostreptocerus* Yu & Cai and is also assigned to Lampriminae based on a suite of traits.

## 1. Introduction

Lucanidae (stag beetles), a small group and one of the earliest branching lineages of Scarabaeoidea, are distributed worldwide except in Antarctica [1,2,3]. The morphological characteristics of most species of stag beetles are relatively complex, and there is not only significant sexual dimorphism but also male polymorphism and different color patterns in the same species [4,5]. Many stag beetles are well known for the greatly enlarged, ornately toothed mandibles of their males that are used in combat with other male opponents during fights to establish dominance [6,7]. The stag beetles occupy a unique niche in coniferous and deciduous forest habitats and are associated with decaying wood and logs. Adults feed on flowers, rotten fruits, sap flows from fluxing trees, and so on, while their larvae live in and feed on decaying wood [6,8]. Therefore, stag beetles are not only popular among insect enthusiasts for their ornamental and collection value, but they also hold significant scientific research value due to their complex morphology, specific behaviors, and wide distribution [9,10]. They serve as an ideal subject for exploring morphological adaptations, sexual selection behaviors, and the mechanisms behind species differentiation and formation.

With more than 110 genera and about 1800 described species [11], Lucanidae currently comprises eight subfamilies, including four extinct (Protolucaninae, Ceruchitinae, Paralucaninae, and Litholampriminae) and four extant ones (Aesalinae, Syndesinae, Lampriminae, and Lucaninae) [12]. With five extant genera (*Dendroblax* White, 1846, *Homolamprima* MacLeay, 1885, *Lamprima* Latreille, 1804, *Phalacrognathus* MacLeay, 1885, and *Streptocerus* Fairmaire, 1850) and nine species, the subfamily Lampriminae is the smallest subfamily of stag beetles, and all extant lamprimines occur exclusively in the Southern Hemisphere [13]. The lamprimines are distributed along the lowland of Southeastern Australia, Lord Howe Island, Norfolk Island, New Zealand, and New Guinea in the Australasian, as well as Southwestern Chile and a small adjacent Argentinian, region in the Neotropics [1]. *Lamprima* includes five species, and the other four genera are monotypic. Recent molecular-based studies indicate the subfamily Lampriminae is monophyletic, while the tribe Lamprimini (*Lamprima* + *Hololamprima* + *Dendroblax* + *Phalacrognathus*) is polyphyletic, where the monotypic tribe Streptocerini (*Streptocerus*) appeared to be a sister group to all *Lamprima* species, with the genus *Phalacrognathus* of Lamprimini falling outside the *Lamprima*-*Streptocerus* clade [1]. 

Fossils belonging to Lampriminae are sparse. To date, only one fossil species of Lampriminae has been formally described. Chalumeau and Brochier [14] described the first known representative of Lampriminae, *Protognathinus spielbergi* Chalumeau & Brochier, 2001, from the Eocene of Messel, Germany. Here, we report one new species of Lampriminae from mid-Cretaceous Burmese (Kachin) amber based on a well-preserved specimen. This new discovery is important for our understanding of the early biodiversity and biogeography of stag beetles.

## 2. Materials and Methods

The amber specimen described here originated from the Hukawng Valley in Tanaing Township, Myitkyina District of Kachin State, northern Myanmar [15], from where a wealth of beetles have been known. The amber piece was grounded and polished in order to make all morphological features accessible for observation. The holotype of *Prostreptocerus burmiticus* Yu & Cai sp. nov. is deposited in the Nanjing Institute of Geology and Palaeontology (NIGP), Chinese Academy of Sciences, Nanjing, China.

Observations and photographs were taken using a Zeiss Axio Imager 2 compound microscope with an AxioCam MRc 5 camera attached (Zeiss, Jena, Germany). The Zeiss Axio Imager 2 microscope was equipped with a mercury lamp and specific filters for DAPI (40,6-diamidino-2-phenylindole), eGFP (enhanced green fluorescent protein), and rhodamine. Photomicrographs with a green background were taken under the eGFP mode, and those with a red background were taken in rhodamine mode. A fluorescence imaging technique is a useful tool to visualize strongly sclerotized inclusions in amber [16]. Confocal images were semi-manually stacked with Helicon Focus 7.0.2 and were further processed in Adobe Photoshop CC to adjust brightness and contrast.

The subfamilial classification system of Lucanidae follows Bouchard et al. [12], and the morphological terminology follows Lawrence et al. [17]. Measurements were taken as follows: body length from anterior margin of clypeus to apex of elytra along the midline; body width as maximum width of body; head length from apex of clypeus to posterior margin of head along the midline; head width as maximum width of head across the eyes; pronotal length from anterior to posterior margin along the midline; pronotal width across the maximum width; elytra length along suture, including scutellum; elytral width as maximum width of elytra combined.

## 3. Results

### Systematic Paleontology

Order Coleoptera Linnaeus, 1758

Suborder Polyphaga Emery, 1886

Superfamily Scarabaeoidea Latreille, 1802

Family Lucanidae Latreille, 1804

Subfamily Lampriminae MacLeay, 1819

Genus *Prostreptocerus* Yu & Cai gen. nov.

Type species *Prostreptocerus burmiticus* Yu & Cai sp. nov., by current designation.

**Etymology.** The Latin term “pro-”, meaning “before” or “previous”, and “*Streptocerus*” after the similar genus of Lampriminae. The gender of the name is masculine. The genus is registered under LSID urn:lsid:zoobank.org:act:B1D1FC98-6404-4635-BC81-D26CAB7AB3A2.

**Figure 1:**
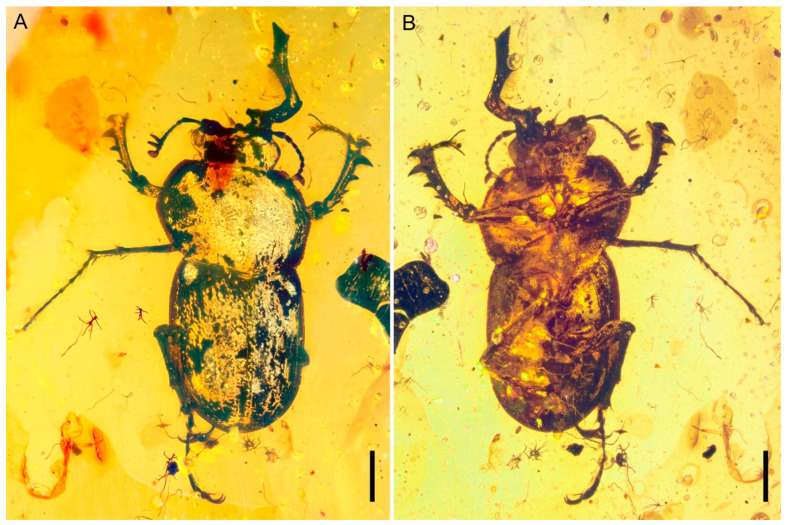
Habitus of *Prostreptocerus burmiticus* Yu & Cai gen. et sp. nov. in mid-Cretaceous amber from Myanmar, holotype (NIGP205392), under incident light: (**A**) Ventral view. (**B**) Dorsal view. Scale bars: 1 mm.

**Figure 2:**
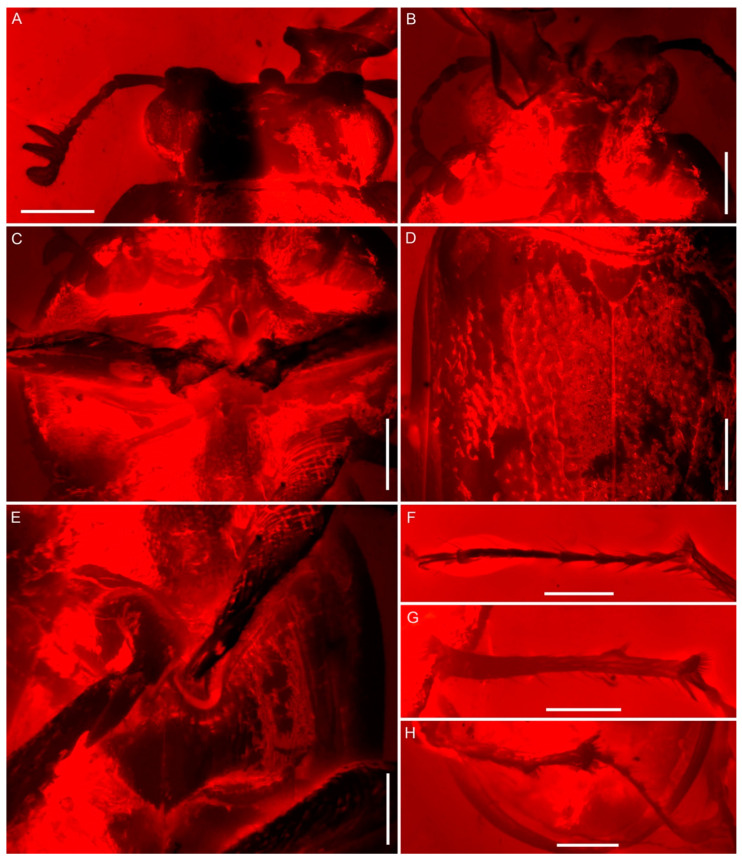
Details of *Prostreptocerus burmiticus* Yu & Cai gen. et sp. nov., under red epifluorescence, holotype (NIGP205392): Dorsal views (**A**,**D**,**F**), ventral views (**B**,**C**,**E**,**G**,**H**). (**A**) Head. (**B**) Head. (**C**) Prothorax. (**D**) elytra. (**E**) Mesothorax. (**F**) Mesotarsus. (**G**) Mesotibiae. (**H**) Metatibiae and mesotarsus. Scale bars: 0.5 mm.

**Figure 3:**
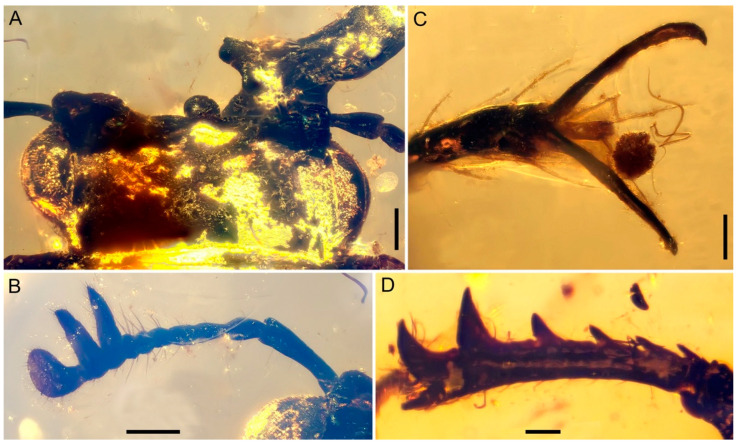
Details of *Prostreptocerus burmiticus* Yu & Cai gen. et sp. nov., under incident light, holotype (NIGP205392): Dorsal views (**A**,**B**,**D**); ventral views (**C**). (**A**) Head. (**B**) Antennae. (**C**) Mesotarsal claw. (**D**) Protibiae. Scale bars: (**B**) 100 μm; others 200 μm.

**Figure 4:**
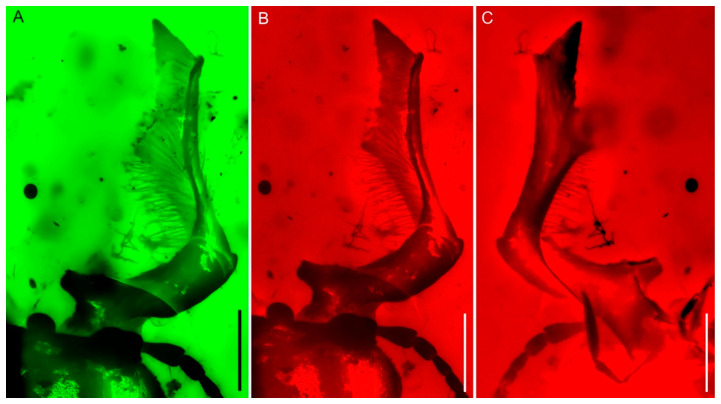
Mandible of *Prostreptocerus burmiticus* Yu & Cai gen. et sp. nov., holotype (NIGP205392): Dorsal views (**A**,**B**); ventral views (**C**). Under green epifluorescence (**A**); under red epifluorescence (**B**,**C**). Scale bars: 0.5 mm.

**Diagnosis.** Body stout and broad (Figure 1); eyes entire, not divided by canthus (Figure 1A, Figure 2A and Figure 3A); antennae partially geniculate (Figure 1, Figure 2A,B and Figure 3B); mandible slightly bent from base to base one-third, then turned upwards about a right angle, concave until to apex (Figure 1 and Figure 4); frons with a pair of well-developed, large protuberances in front of eye (Figure 1A, Figure 2A and Figure 3A); lateral pronotal carinae crenulate (Figure 1); procoxal process strongly narrowed (Figure 1B and Figure 2C); protibiae gradually expanded from base to apex (Figure 1 and Figure 3D); protibial teeth increasing gradually in size from base to apex (Figure 1 and Figure 3D); elytral disc coarsely and sparsely punctated, without distinct rows or striae, with a pair of large tubercles on the humeri produced anteriorly (Figure 1A and Figure 2D); pretarsal claws large and simple, arolia with two bristles apically (Figure 3C). 

*Prostreptocerus burmiticus* Yu & Cai sp. nov.

(Figure 1, Figure 2, Figure 3 and Figure 4)

**Etymology.** The specific epithet “*burmiticus*” refers to the occurrence of the fossil in burmite (Burmese amber). The species is registered under LSID urn:lsid:zoobank.org:act:8205D1F6-4CD2-4384-B700-633B53FC5EE7.

**Material.** Holotype: NIGP205392, male, completely preserved adults. The specimen is permanently housed in the Nanjing Institute of Geology and Paleontology, Chinese Academy of Sciences (CAS), Nanjing, China. Preserved in Burmese amber; absolute age: 98.79 ± 0.62 Ma [18,19], established by the U-Pb (uranium-lead) dating of zircons from the associated matrix of the unprocessed amber.

**Diagnosis.** As for the genus (vide supra).

**Description.** Male (based on shape and size of mandible). Body oval-shaped, slightly flattened, 2.0 times as long as wide; length 6.07 mm (measured from apex of clypeus to apex of elytra), 8.04 mm (including mandibles), mandibles 25% of overall length; pronotum slightly broader than elytra. Color dark brown. Dorsum apparently glabrous, sparsely punctate, punctures on pronotum and elytra large. 

Head prognathous, not deeply inserted into prothorax, and not abruptly constricted posteriorly; transverse, length slightly less than half width, 0.78 mm long (measured from apex of clypeus to anterior margin of pronotum) and 1.78 mm in width at maximum, broadest at eyes; dorsum coarse, anterior margin truncate. Eyes distinctly protuberant, large, and oval; ocular canthus probably absent. Frontoclypeal suture absent. Frons with a pair of well-developed, large protuberances in front of eye. Labrum not fused to clypeus, transverse, convex at the middle of anterior margin. Antennae moderately long, weakly geniculate, with 10 antennomeres; three-segmented loose and pectinate club, which is strongly asymmetrical and covered with pubescence; scape elongate, more than four times as long as wide and about three times as long as pedicel, slightly arched, widened distally; pedicel subcylindrical, thinner than scape, less than two times as long as wide; antennomere 3 elongate, thinner and slightly shorter than pedicel, slightly widened distally; antennomeres 4–6 each longer than wide, subequal with antennomere 3 and slightly wider than antennomere 3, almost of same width and length; antenomere 7 cylindrical, shorter than antennomere 6; antennomeres 8–9 transverse, subcylindrical; antennomere 10 rounded apically, apparently longer and thinner than antennomere 9. Mandibles extremely developed, obviously longer than head; slightly bent from base to base one-third, and then turned upwards at about a right angle, concave until to apex; with an obtuse tooth on inner edges of base, a sharp tooth at the apical one-third and apical, respectively; multi-small teeth along the inner edge from apical one-third to apex; with dense long setae erected on inner surfaces. Maxillary palpus long, with apical palpomere longest, fusiform. Mentum transverse, apical labial palpomere fusiform. Gular sutures well separated. 

Pronotum length 2.10 mm, width 3.07 mm; about 0.7 times as long as wide, and widest at about middle; obviously wider than head, base as wide as combined elytral bases; disc slightly convex, densely punctured, with several longitudinal wrinkles near the lateral margin; sides moderately rounded, not explanate. Lateral pronotal carinae complete, simple, and crenulate, without raised margin; anterior angles slightly produced forward and posterior angles obtuse; anterior margin nearly truncate, posterior margin slightly biemarginate, anterior margin subequal to basal margin. Pronotal hypomeron with closely recumbent setae in posterior half. Prosternum biconcave, with densely erect setae at the anterior margin. Prosternal process narrow. Procoxae large, transverse, and subcontiguous, projecting below prosternum; trochantin concealed. Procoxal cavities strongly transverse, narrowly separated, and broadly closed externally. 

Scutellar shield well developed, nearly triangular, and posteriorly narrowly rounded, with sparse punctures. Elytra length 3.19 mm, combined width 2.65 mm, about 1.2 times as long as width and 1.5 times as long as pronotum; base distinctly arched to accommodate base of pronotum; lateral margin smooth and slightly rounded; broadest at anterior two-fifths and then contracted to humeri and conjointly rounded apices; epipleura narrow and complete, broadest at humeri, and then slightly contracted to apices; disc coarse and sparsely punctate, without distinct rows or striae, and with a pair of large tubercles on the humeri that produced anteriorly. Mesocoxae slightly oblique. Mesocoxal cavities narrowly separated. Mesoventrite, mesanepisternum and mesepimeron with densely decumbent setae. Metaventrite transverse and slightly convex, without postcoxal lines; metakatepisternal suture absent; discrimen complete; exposed portion of metanepisternum moderately long and narrow. Metacoxae strongly transverse and subcontiguous, extending laterally to meet elytra. 

Legs relatively long, densely setose. Profemur expanded and flattened. Protibia slightly expanded apically with one stout curved apical tooth and five well-spaced teeth along outer edge gradually increasing in size to outer apical angle; inner lateral margin with an apical spur. Meso- and metafemur robust, fusiform. Meso- and metatibiae with one subtriangular tooth near the apical one-third along outer edge, a pair of apical spurs, a circle of short denticles along apical edge and several long denticles along lateral margin. Tarsi 5-5-5; tarsomeres slender, simple; tarsomeres I–II shortest and subequal in length; tarsomeres 3–4 slightly longer than tarsomeres 1–2, and subequal in length; tarsomere 5 longest, slightly longer than basal four tarsomeres combined. Pretarsal claws large and simple; rod short, two long bristles arranged on either side of its apex.

Abdomen with five free ventrites, densely setose. 

## 4. Discussion

**Systematic position of *Prostreptocerus* Yu & Cai gen. nov.**

*Prostreptocerus* Yu & Cai can be classified into Lucanidae based on a combination of the following characteristics: mandible extremely developed; antennae partially geniculate, 10-segments with 3-segmented club; antennal club loose, tomentose; scape long, slender; mentum entire; pronotum large; procoxae very large, transverse, projecting below the prosternum and with posteriorly closed cavities; protibiae with several teeth on the outer edge and a single apical spur; mesocoxal cavities open laterally; tarsi 5-5-5, with empodium bisetose; and abdomen with five ventrites.

*Prostreptocerus* Yu & Cai is similar to the extinct litholamprimins in the shape of the body and antennae, as well as having exaggerated mandibles [20,21]. However, it differs in several key aspects: *Prostreptocerus* Yu & Cai has a smaller body size, procoxae subcontiguous and prosternal process narrow. 

*Prostreptocerus* Yu & Cai is placed in Lampriminae and is distinguished from other subfamilies in Lucanidae via a combination of the following characteristics: body stout and broad; antennae partially geniculate; eyes entire and not divided by canthus; prementum situated near apex of mentum; prosternal process strongly narrowed, not expanded anteriorly and not reaching metaventrite; procoxae subcontiguous; elytra not conspicuously parallel-sided, their width together about 0.8 times elytral length; elytral surface lacks dense vestiture, with sparse simple punctures; protibiae somewhat fossorial, gradually expanded from base to apex; protibial teeth increasing gradually in size from base to apex; arolia with two bristles apically; abdominal ventrites freely articulated. 

There are five extant genera in Lampriminae. The New Zealand genus *Dendroblax* is unmistakably different from *Prostreptocerus* Yu & Cai, with a very convex, scarabaeid-like body, small mandibles in both sexes, strongly fossorial legs, and dense, long setae on the underside of the body [13]. The northern Australian endemic *Phalacrognathus* is relatively easily distinguished from *Prostreptocerus* Yu & Cai by its large size and apparently smooth body; mandible straight and long, apically bidentate in males; pronotum sides curved anteriorly and crenulated posteriorly; and elytra outer margin crenulated anteriorly. Most species of *Lamprima* can easily be distinguished from *Prostreptocerus* Yu & Cai by the male protibiae with spur expanded as a flat blade, the clubbed antennomeres entirely and densely setose and often closely appressed, and elytra surface always with finely microreticulate microsculpture [22]. However, *L. imberbis* Carter, 1926 is unusual in *Lamprima*, with its male protibial spur not expanded, mandibles lacking internal setae, and the elytra broadly explanate. By comparing the new genus with *L. imberbis*, there are apparent differences in the structure of mandibles (glabrous or almost so on inner surfaces in *L. imberbis* and with dense long setae erected on inner surfaces in *Prostreptocerus* Yu & Cai), protibial teeth (external margin with four teeth in *L. imberbis* and five teeth in *Prostreptocerus* Yu & Cai), and pronotal shape (slightly narrower than elytra in *L. imberbis* vs. base as wide as combined elytral bases in *Prostreptocerus* Yu & Cai). *Prostreptocerus* Yu & Cai resembles the Australian endemic *Homolamprima*, but *Homolamprima* differs from it due to the following characteristics: mandibles short and apically bidentate, pronotal anterior margin narrowed, apex of prosternal process elevated, mesometaventrite junction anteriorly bilobed [23]. The South American genus *Streptocerus* is most similar to *Prostreptocerus* Yu & Cai but differs from it and all other Lampriminae due to the four-segmented antennal club [24]. The narrow head, general form, and the shape of mandible of the new genus show an affinity with *Streptocerus*, but differ from *Streptocerus* due to mandible with dense erect setae on inner surfaces at middle, antennae partially geniculate, scape relatively short, eyes large and prominent, elytral surface with sparse simple punctures.

The mandibles of *Prostreptocerus* Yu & Cai probably remind us of some members in *Syndesus* MacLeay, 1819, of the subfamily Syndesinae. In fact, *Syndesus* is immediately differentiable by its general habitus of cylindricus and pronotum with a median tubercle [13]. *Prostreptocerus* Yu & Cai differs markedly from the poorly preserved *Protognathinus* Chalumeau & Brochier, 2001 [14], which has recently been classified within the subfamily Lampriminae [25], primarily based on the straight or subgeniculate antennae, antennal club with three antennomeres, and largely tridentate protibiae [1]. In the original paper, the description based on the illustration was too simplistic, and the characters that used to place the fossil species *P. spielbergi* into the “Chiasognathinae” are not diagnostic, which are also present in other lucanids. Moreover, the Chiasognathinae is no longer a valid taxonomic group. Therefore, we follow the systematic placements of *Protognathinus* by Paulsen [25]. *Protognathinus* is characterized by its larger size, relatively short scape, and trapezoidal pronotum, contrasting with the features of *Prostreptocerus* Yu & Cai.

Six genera are known from the same deposit, including the new genus [26,27,28,29,30]; here, a key to the known genera of the Lucanidae in mid-Cretaceous Kachin amber is as follows:

**Key to known genera of the Lucanidae in mid-Cretaceous Kachin amber**

1. Antenna completely geniculate……………*Anisoodontus* Wu, Tang & Peng, 2022

- Antenna only partially or not geniculate………………………………………………2

2. Elytra tuberculate in posterior half………………………*Oncelytris* Li & Cai, 2023

- Elytra without distinct tubercles in posterior half……………………………………3

3. Pronotum and elytra covered with flattened scales ……………………………………

………………………………………………………*Protonicagus* Cai, Yin, Liu & Huang, 2017

- Pronotum and elytra without scales……………………………………………………4

4. Lateral pronotal margins smooth…………………*Cretognathus* Yamamoto, 2023

- Lateral pronotal margins slightly serrate………………………………………………5

5. Mandible length subequal to head……*Electraesalopsis* Bai, Zhang & Qiu, 2017

- Mandible obviously longer than head…………………*Prostreptocerus* Yu & Cai gen. nov.

**Systematic position of *Electraesalopsis* Bai, Zhang & Qiu, 2017**

Qiu et al. [27] described the genus *Electraesalopsis* Bai, Zhang & Qiu, 2017, from Kachin amber, but its systematic position within Lucanidae was uncertain. Consequently, the original authors classified *Electraesalopsis* as Lucanidae *incertae sedis*. Here, we suggest further placing it in the subfamily Lampriminae based on a detailed list of characteristics. According to the key of the subfamily of Lucanidae [13], the subfamily Lampriminae is characterized by the following combination of characteristics: (1) body stout and broad; (2) integument of dorsal surface uniformly chocolate brown or metallic green; (3) elytra not conspicuously parallel-sided, and their width together about 0.8 times the elytral length; (4) mandibles of females very deep and with a small inwardly or forwardly directed ventral tooth at or near base; (5) protibiae somewhat fossorial, gradually expanded from base to apex; (6) protibial teeth increasing gradually in size from base to apex of segment; (7) arolia with more than two setae or bristles apically; (8) elytral vestiture consists of simple setae that are sometimes compressed apically, each seta with a large pore near its base. Unfortunately, these characteristics are poorly preserved in most fossil specimens, including *Electraesalopsis*. Based on the visible external morphological characteristics that match Lampriminae, we can assign it to this subfamily according to the following characteristics: body stout and broad, elytra not conspicuously parallel-sided, mandibles very deep and seems with a small inwardly directed ventral tooth near base; protibiae somewhat fossorial, gradually expanded from base to apex; protibial teeth increasing gradually in size from base to apex of segment. Extant lamprimines have entire eyes and narrowly separated procoxae, and *Electraesalopsis* shares these characteristics, which supports its placement within Lampriminae. The structure of the elytral surface in lamprimines is extremely distinctive and unique within Lucanidae. The surface lacks dense vestiture, having sparse, simple, undivided linear setae or scales instead, each with a large pore close to its base (but visible only under a scanning electron microscope) [31]. In *Electraesalopsis*, elytra apparently lack dense vestiture, which is the same as for lamprimines, but other detailed characteristics on the elytral surface are difficult to observe. In the original discussion, *Electraesalopsis* is excluded from Lampriminae based on its smaller body size, even and dense punctures on the body surface [27]. In fact, the body size probably cannot be a diagnostic character because even the same species of lucanids could vary greatly. In addition, punctures on the body surface cannot be used as a distinguishing characteristic of *Electraesalopsis* from Lampriminae because similar punctures are also present in some genera of Lampriminae, such as *Lamprima* and *Dendroblax*. Although the ventral side of the specimen is hazy, it can be seen from the description, diagnosis, and illustrations provided by the authors, meaning that *Electraesalopsis* can easily be distinguished from Aesalinae by the latter subfamily having elytral vestiture that always includes scales, the first two ventrites are connate, and the prosternal process is broad. *Electraesalopsis* could also be confidently distinguished from Syndesinae, which usually has an elongated, cylindrical body. It can be easily separated from Lucaninae due to its weakly geniculate antennae (compared with the geniculate antennae of Lucaninae) and entire eyes (rather than the partially or completely divided eyes of Lucaninae). Therefore, we recommend that *Electraesalopsis* should be placed in Lampriminae. 

On the other hand, *Electraesalopsis* is similar to *Prostreptocerus* Yu & Cai gen. nov. in its general habitus, including some detailed characteristics, such as the slightly serrate lateral margin of the pronotum and the rod with two long bristles. However, they can be distinguished from each other according to the shape of the pronotum and the presence of protuberances on the head and elytra. Given their shared characteristics, as well as similar deposits, ages, and preservation, we propose a close relationship between them. The smaller mandibles of *Electraesalopsis* suggest that the holotype is likely a female.

**Biogeographic implications**

A total of 36 fossil stag beetle species have been described from various deposits worldwide. A catalogue of these species is provided in Table 1, which is primarily based on several key references [28,30,32,33].

These species are distributed in 26 genera of all eight subfamilies, summarized as follows: Middle Jurassic (China, 1 sp.); Upper Jurassic (Mongolia, 2 spp.); Lower Cretaceous (China, 7 spp.); Lower Cretaceous (Russia, 3 spp.); mid-Cretaceous (Myanmar amber, 10 spp.); Upper Cretaceous (Kazakhstan, 1 sp.); Eocene (Germany, 1 sp.); Eocene (Czech Republic, 1 sp.); Eocene (Baltic amber, 3 spp.); Late Eocene (Dominican amber, 1 sp.); Oligocene (Germany, 2 spp.); Oligocene (USA, 2 spp.); Oligocene (Russia, 1 sp.); Miocene (France, 1 sp.). Despite the controversies on the origin of the West Burma block, all the Mesozoic lucanids are distributed in the Northern Hemisphere, which belonged to Laurasia during that time, whereas there is worldwide distribution in the Cenozoic. It suggests that Lucanidae probably originated in Laurasia and were then dispersed to the Gondwana, subsequently forming the world distribution pattern we see since the Cenozoic. 

There are 10 records of lucanids from Burmese amber, which indicates potentially a high number of species and morphological diversity in a tropical forest in mid-Cretaceous Myanmar. The lucanid fossil described herein from mid-Cretaceous Burmese amber likely represents the oldest record of Lampriminae, highlighting the ancient origin of this subfamily. This fossil evidence aligns with the hypothesis of an older origin for Lampriminae among the lucanid subfamilies, as suggested by molecular data [1]. While recent lamprimines are restricted to the Southern Hemisphere, fossil lamprimines have been found in Eocene Germany (*Protognathinus spielbergi*) and mid-Cretaceous Myanmar (*Prostreptocerus burmiticus* Yu & Cai gen. et sp. nov.). This indicates that lamprimines were probably more widespread than they are at present. The mid-Cretaceous presence of *Prostreptocerus* Yu & Cai in present-day Southeast Asia and its classification within Lampriminae, for which the genera are exclusively found in austral regions, suggest that ancestral Lampriminae existed on Gondwanaland. Significant extinction events likely occurred in Laurasia after the Eocene, leading to the current restricted distribution of Lampriminae. In recent years, an increasing number of taxa have been discovered in Kachin amber, with their closest extant relatives being restricted to the Southern Hemisphere [34,35,36,37]. These findings have sparked significant interest among researchers, leading to detailed discussions on the biogeographical implications. Such studies have provided further evidence of the affinities between the paleoentomofauna of Myanmar and Gondwanan regions [38,39].

**Sexual dimorphism and fierce fighting in fossil lucanids**

Most adult lucanids exhibit pronounced sexual dimorphism, with males possessing well-developed and variable mandibles used for biting and fighting fiercely for access to mates [40]. In NIGP205392, the mandible length is significantly longer than the head, constituting about 25% of the overall body length. This characteristic, along with its particular shape, indicates that the specimen is a male. Research on stag beetle battle behavior and associated anatomical adaptations has shown that beetles with longer mandibles have a higher chance of winning battles. Mandible length is more crucial than body length for success in fights, as longer mandibles provide better reach to grasp and detach a rival’s legs or even lift a rival from the ground to allow for more forceful bites [40]. *Prostreptocerus burmiticus* Yu & Cai has a single, extremely developed mandible that is preserved, indicating its capability for fighting. Additionally, several scratches on the pronotum, likely caused by a sharp structure and the missing mandible, may be evidence of a fierce battle. In stag beetle battles, it is crucial to grasp an opponent’s legs, haul them, and wrestle to dislodge each other. Maintaining balance while grabbing or lifting the opponent and avoiding being dislodged and lifted are equally important. Mandibles are used for grasping, while tarsal claws aid in balance. The claws in this specimen become more curved from the protarsi to the metarsi, with the hind leg claws being the most curved. This finding aligns with the findings of Goyens et al. [40], suggesting that hind leg claws are crucial for lifting. 

Determining the sex of fossil specimens can be challenging due to the often unexposed or poorly preserved genital organs. However, other sexually dimorphic characteristics, such as mandibles or antennal clubs, can be used for identification. Recent fossil studies have demonstrated similar sexual dimorphism and notable features [41,42,43]. For fossil lucanids, mandible size and shape are particularly useful for sex identification [21,26,28,33]. No lucanids from Kachin amber have shown distinctly male mandibles [30]. These findings, along with the newly described species, offer additional evidence of sexual dimorphism among beetles in the Mesozoic.

## 5. Conclusions

We report a new lucanid fossil, *Prostreptocerus burmiticus* Yu & Cai gen. et sp. nov., based on a well-preserved specimen in mid-Cretaceous amber from northern Myanmar. *Prostreptocerus* Yu & Cai, as one of the earliest diverging lamprimines, provides evidence that this lineage has existed for at least 99 million years. Additionally, *Electraesalopsis*, previously classified as Lucanidae *incertae sedis*, is now assigned to Lampriminae based on shared morphological features. While extant lamprimines are confined to the Southern Hemisphere, fossil records from Laurasia suggest that ancestral Lampriminae existed on Gondwanaland, with significant extinction events in Laurasia after the Eocene. The well-developed mandibles and curved claws of *Prostreptocerus* Yu & Cai likely indicate its strong fighting ability, supporting the presence of sexual dimorphism and potential battle behavior in Mesozoic lucanids.

## Figures and Tables

**Table 1:** The catalogue of known fossil species of Lucanidae.

Subfamily	Genera	Species	Distribution
Aesalinae MacLeay, 1819	*Cretaesalus* Nikolajev, 1993	*Cretaesalus ponomarenkoi* Nikolajev, 1993	Kazakhstan (Upper Cretaceous)
	*Cretognathus* Yamamoto, 2023	*Cretognathus minutissimus* Yamamoto, 2023	Myanmar amber (mid-Cretaceous)
	*Juraesalus* Nikolajev et al., 2011	*Juraesalus atavus* Nikolajev et al., 2011	China (Middle Jurassic)
	*Oncelytris* Li & Cai, 2023	*Oncelytris esquamatus* Li & Cai, 2023	Myanmar amber (mid-Cretaceous)
	*Protonicagus* Cai et al., 2017	*Protonicagus mandibularis* Yamamoto, 2023	Myanmar amber (mid-Cretaceous)
		*Protonicagus tani* Cai et al., 2017	Myanmar amber (mid-Cretaceous)
	*Sinaesalus* Nikolajev et al., 2011	*Sinaesalus curvipes* Nikolajev et al., 2011	China (Lower Cretaceous)
		*Sinaesalus longipes* Nikolajev et al., 2011	China (Lower Cretaceous)
		*Sinaesalus tenuipes* Nikolajev et al., 2011	China (Lower Cretaceous)
	genus *incertae sedis*	Ceratognathini gen. et sp. indet. 1	Myanmar amber (mid-Cretaceous)
		Ceratognathini gen. et sp. indet. 2	Myanmar amber (mid-Cretaceous)
Lampriminae MacLeay, 1819	*Electraesalopsis* Bai et al., 2017	*Electraesalopsis beuteli* Bai et al., 2017	Myanmar amber (mid-Cretaceous)
	*Protognathinus* Chalumeau & Brochier, 2001	* *Protognathinus spielbergi* Chalumeau & Brochier, 2001	Germany (Eocene)
	*Prostreptocerus* Yu & Cai gen. nov.	*Prostreptocerus burmiticus* Yu & Cai sp. nov.	Myanmar amber (mid-Cretaceous)
Lucaninae Latreille, 1804	*Anisoodontus* Wu et al., 2022	*Anisoodontus qizhihaoi* Wu et al., 2022	Myanmar amber (mid-Cretaceous)
		*Anisoodontus xiafangyuani* Wu et al., 2022	Myanmar amber (mid-Cretaceous)
	*Dorcus* Macleay, 1819	*Dorcus primigenius* Deichmüller, 1881	Czech Republic (Eocene)
	*Lucanus* Goldfuss, 1831	* *Lucanus fossilis* Wickham, 1913	USA (Oligocene)
	*Miocenidorcus* Riou, 1999	*Miocenidorcus andancensis* Riou, 1999	France (Miocene)
	*Platycerus* Geoffroy, 1762	* *Platycerus sepultus* Germar, 1837	Germany (Oligocene)
		*Platycerus zherichini* Nikolajev, 1990	Russia (Oligocene)
	*Prolucanus* Qi et al., 2022	*Prolucanus beipiaoensis* Qi et al., 2022	China (Lower Cretaceous)
	*Succiniplatycerus* Nikolajev, 1990	*Succiniplatycerus berendti* (Zang, 1905)	Baltic amber (Eocene)
Syndesinae MacLeay, 1819	*Ceruchus* Wickham, 1911	*Ceruchus fuchsii* Wickham, 1911	U.S.A. (Oligocene)
	*Prosinodendron* Bai et al., 2012	*Prosinodendron krelli* Bai et al., 2012	China (Lower Cretaceous)
	*Syndesus* Woodruff, 2009	*Syndesus ambericus* Woodruff, 2009	Dominican amber (Late Eocene)
Ceruchitinae Nikolajev, 2006	*Ceruchites* Statz, 1952	*Ceruchites hahnei* Statz, 1952	Germany (Oligocene)
Litholampriminae Nikolajev & Ren, 2015	*Litholamprima* Nikolajev & Ren, 2015	*Litholamprima longimana* Nikolajev & Ren, 2015	China (Lower Cretaceous)
		*Litholamprima qizhihaoi* Jiang et al., 2022	China (Lower Cretaceous)
Paralucaninae Nikolajev, 2000	*Paralucanus* Nikolajev, 2000	*Paralucanus mesozoicus* Nikolajev, 2000	Mongolia (Upper Jurassic)
Protolucaninae Nikolajev, 2007	*Protolucanus* Nikolajev, 2007	*Protolucanus jurassicus* Nikolajev, 2007	Mongolia (Upper Jurassic)
Subfamily *incertae sedis*	*Cretolucanus* Nikolajev, 2007	*Cretolucanus longus* Nikolajev, 2007	Russia (Lower Cretaceous)
		*Cretolucanus ordinarius* Nikolajev, 2007	Russia (Lower Cretaceous)
		*Cretolucanus sibericus* Nikolajev, 2007	Russia (Lower Cretaceous)
	*Dorcasoides* Motschulsky, 1856	*Dorcasoides bilobus* Motschulsky, 1856	Baltic amber (Eocene)
	*Paleognathus* Waga, 1883	*Paleognathus succini* Waga, 1883	Baltic amber (Eocene)

Note: We followed the systematic placements based on the latest references. “*” indicates that the species is doubtful.

## Data Availability

The original contributions presented in the study are included in the article, further inquiries can be directed to the corresponding authors.
